# Protocol for the formative phase of a trial (SHE-CAN) to test co-designed implementation strategies for HPV-based cervical screening among vulnerable women in two diverse settings in India

**DOI:** 10.1186/s43058-023-00436-0

**Published:** 2023-06-08

**Authors:** Anu Mary Oommen, Partha Basu, Anne George Cherian, Eric Zomawia, Ravikumar Manoharan, Ruby Angeline Pricilla, Vidhya Viswanathan, Brian Oldenburg, Sujha Subramanian, David Hawkes, Marion Saville, Julia M. L. Brotherton, on behalf of the SHE-CAN collaborators, Abraham Peedicayil, Jeremy L. Pautu, Sangzuala, Vanremmawii, Evelyn V. L. Hmangaihzuali, Lalropuii, Pravin Singarayar, Kuryan George, J. Grace Rebekah, Tarun George, Jasmine Prasad, Tobey Marcus, Anitha Thomas, Vinotha Thomas, Dhanya S. Thomas, Sherin Daniel, Kripa M. Varghese, Vinod J. Abraham, Divya E. Muliyil, Rajesh Kannangai, Priya Abraham, Anuradha Rose, Shalini Jeyapaul, Tabeetha Malini, Thomas S. Ram, Neenu O. John, D. Priya Ranjani, K. Kavitha, G. Meenatchi, John Paul, Pavan K. Mukherjee, Sasikala Umesh, K. R. John, Claire Nightingale, Sumit Kane, Maleeha Ashfaq, Arunah Chandran, Isabel Mosquera, Richard Muwonge, Andre Carvahlo

**Affiliations:** 1 The University of Melbourne, Melbourne, Australia; 2 Christian Medical College, Vellore, Tamil Nadu India; 3 International Agency for Research on Cancer, Lyon, France; 4 Population Based Cancer Registry, Aizawl, Mizoram India; 5 The Tribal Health Initiative, Sittilingi, Dharmapuri, Tamil Nadu India; 6 Directorate of Public Health and Preventive Medicine, Government of Tamil Nadu, Chennai, India; 7 Baker Heart and Diabetes Institute, Melbourne, Australia; 8 Research Triangle Institute: RTI International, Waltham, MA USA; 9 Australian Centre for Prevention of Cervical Cancer, Melbourne, Australia; 10 Formerly Australian Centre for Prevention of Cervical Cancer, Melbourne, Australia

**Keywords:** HPV, Cervical, Screening, Co-design, Implementation, India

## Abstract

**Background:**

In view of the WHO’s call for the elimination of cervical cancer as a public health problem, and current low screening coverage, Indian policy makers need evidence on how to effectively implement cervical screening programmes, ensuring equity in access.

Our study will follow the INSPIRE implementation framework to co-design and test HPV-based screening approaches in two states of India with different health system organisation, based on understanding the status of screening as currently implemented, readiness and challenges to transition to HPV-based screening, and preferences of key stakeholders. Here, we describe our protocol for the formative phase of the study (SHE-CAN).

**Methods:**

The study population includes women from vulnerable populations, defined as residents of tribal areas, rural villages, and urban slums, in the states of Mizoram and Tamil Nadu. The baseline assessment will use mixed methods research, with desktop reviews, qualitative studies, and surveys. A capacity assessment survey of screening and treatment facilities will be done, followed by interviews with healthcare providers, programme managers, and community health workers. Interviews will be conducted with previously screened women and focus group discussions with under and never-screened women and community members. Stakeholder workshops will be held in each state to co-design the approaches to delivering HPV-based screening among 30–49-year-old women.

**Discussion:**

The quality and outcomes of existing screening services, readiness to transition to HPV-based screening, challenges in providing and participating in the cervical cancer care continuum, and acceptability of screening and treatment approaches will be examined. The knowledge gained about the current system, as well as recognition of actions to be taken, will inform a stakeholder workshop to co-design and evaluate implementation approaches for HPV-based screening through a cluster randomised implementation trial.

Contribution to literature

- The protocol for the SHE-CAN study describes the steps involved in developing co-designed implementation approaches for HPV-based cervical screening for women from hard to reach populations in India.
- This protocol is an example of implementation research methodology based on the recent INPIRE framework that describes the formative phase of situational analysis of the current screening programme and co-design of HPV-based screening approaches, prior to testing out the co-designed approaches in a cluster randomised trial, with finalisation of trial and comparator arms to be done through the formative co-design process.
- This protocol adds to the literature on approaches to assessing and developing complex interventions.

## Background

The World Health Organization (WHO) launched a global strategy in 2020 for the elimination of cervical cancer as a public health problem, defined as the reduction of global incidence to < 4 per 100,000 women, thus averting more than 70 million cervical cancer deaths over the next 100 years [1, 2]. With 123,907 new cases and 77,348 deaths reported in 2020, India contributed nearly a quarter of the global burden of cervical cancers [3]. The National Cancer Registry Programme reported the age-standardised incidence rate in India as 18.0 per 100,000 in 2020 [3], with high variability in trends across different states [4, 5]. Mizoram was the state with the highest annual percent increase in age-adjusted cervical cancer incidence (2.1% increase between 2004 and 2016), while the state of Tamil Nadu had the second highest DALY (disability-adjusted life year) rate [5], but recorded the highest rate of decline in incidence during the same period (3.5% annual percent change) [4].

The National Programme for Prevention and Control of Cancers, Diabetes, Cardiovascular Diseases and Stroke (NPCDCS), launched in India in 2010, recommended offering cervical screening using Visual Inspection with Acetic Acid (VIA) for women aged 30–65 years every 5 years, with nationwide scale up planned in 2016 [6, 7].

Despite provisions of community-based screening, the National Family Health Survey (NFHS)-5 (2019–2021) found that only 1.9% (urban 2.2%, rural 1.7%) of the sampled Indian women aged 30–49 years had ever undergone cervical screening, which was far below the WHO target of 70% coverage [8]. In Tamil Nadu, a state with a relatively well functioning NPCDCS programme, only 9.8% (urban 10.0%, rural 9.6%) of women 30–49 years reported ever having been screened for cervical cancer; in Mizoram, a state in the hard-to-reach north-east part of India, only 6.9% of women (urban 9.4%, rural 3.3%) have ever been screened [9].

The extremely low screening coverage highlights the difficulties in scaling up VIA-based screening in low- and middle-income countries (LMICs) like India, due to challenges such as high training requirements, subjectivity of the test, variable sensitivity, and difficulty in ensuring quality. Population level barriers, such as poor awareness, hesitancy of asymptomatic women to undergo a pelvic examination, and structural barriers to access services further compound the problem [10–12].

HPV detection-based screening, recommended by the WHO [2] as the optimal approach for cervical cancer elimination, has been shown to be feasible in LMICs, through demonstration projects, including at scale, in several diverse regions in Asia [13], Africa [14], Asia-Pacific [15], and the Americas [16]. A distinct advantage of HPV-based screening using a PCR-based test is that the test performed on a sample self-collected from the upper vagina is as sensitive as one performed on samples collected from the cervix by a provider, for detecting cervical intraepithelial neoplasia grade 2 or worse (CIN 2+) [17]. The ability of women to collect their own specimens overcomes some of the barriers to screening, with higher participation shown in multiple settings, compared to clinician-collected sample [17–23].

Despite compelling evidence of the effectiveness of HPV-based screening, the adoption and scaling up of quality-assured HPV-based screening is yet to occur in most LMICs. This is due to the current high cost of clinically validated HPV tests, complexities of setting up a laboratory, and challenges in managing the logistics of sample collection, transport, and report delivery. In India, a feasible and sustainable model of service delivery for self-collection based HPV screening is needed, as is demonstration of a clear logistic, economic, and health benefit of such a service model over ongoing VIA-based screening. Unlike the modelling exercise performed to inform the WHO recommendations, an Indian study did not find 5-yearly HPV screening to be more cost-effective than 5-yearly VIA, possibly because of the difference in assumptions of test characteristics [24].

Early gains in reduction of incidence and mortality from cervical cancer in India will be realised through successful introduction and scale up of HPV-based screening, together with treatment, for all sections of the population, ensuring equitable access to evidence-based strategies. Co-designing along with local stakeholders a contextually appropriate service delivery model to extend HPV screening among vulnerable women and gathering local evidence on feasibility, acceptability, effectiveness, sustainability, and economic benefit of this new model is the focus of our implementation research, the objectives and methodology of which are described in the next sections.

## Objectives of our study and underlying frameworks

SHE-CAN (Self-collected HPV Evaluation for the Prevention of Cervical CANcer) is an implementation research study supported by the Global Alliance for Chronic Disease (GACD) and National Health and Medical Research Council of Australia (NHMRC). The Indian partners include not-for-profit health care organisations like Christian Medical College Vellore (CMC) in Vellore district and the Tribal Health Initiative (THI) hospital in Dharmapuri district, in partnership with the Directorate of Public Health and Preventive Medicine (DPHPM), in the state of Tamil Nadu, and the state government’s Population Based Cancer Registry (PBCR) in Mizoram. International partners include researchers from the Australian Centre for Prevention of Cervical Cancer (ACPCC), International Agency for Research on Cancer (IARC) (France), Baker Heart and Diabetes Institute (Australia) and the Research Triangle Institute (RTI International) (USA). The project will draw on international and local learnings and will engage with stakeholders in India to co-design, implement, and evaluate multiple implementation approaches to screen vulnerable populations (tribal, rural, and urban slum dwellers), in Tamil Nadu and Mizoram, with an HPV test.

Implementation approaches will consider the possible roles of self-collection, using community health workers for educating and navigating women, emerging point-of-care HPV tests, screen and treat algorithm to manage HPV positive women, and use of digital technologies to support women complete the screening (and, where indicated, treatment) pathway. Among the implementation research outcomes, cost effectiveness analysis will consider twice in a lifetime HPV test vs five yearly VIA screening.

**Fig. 1:**
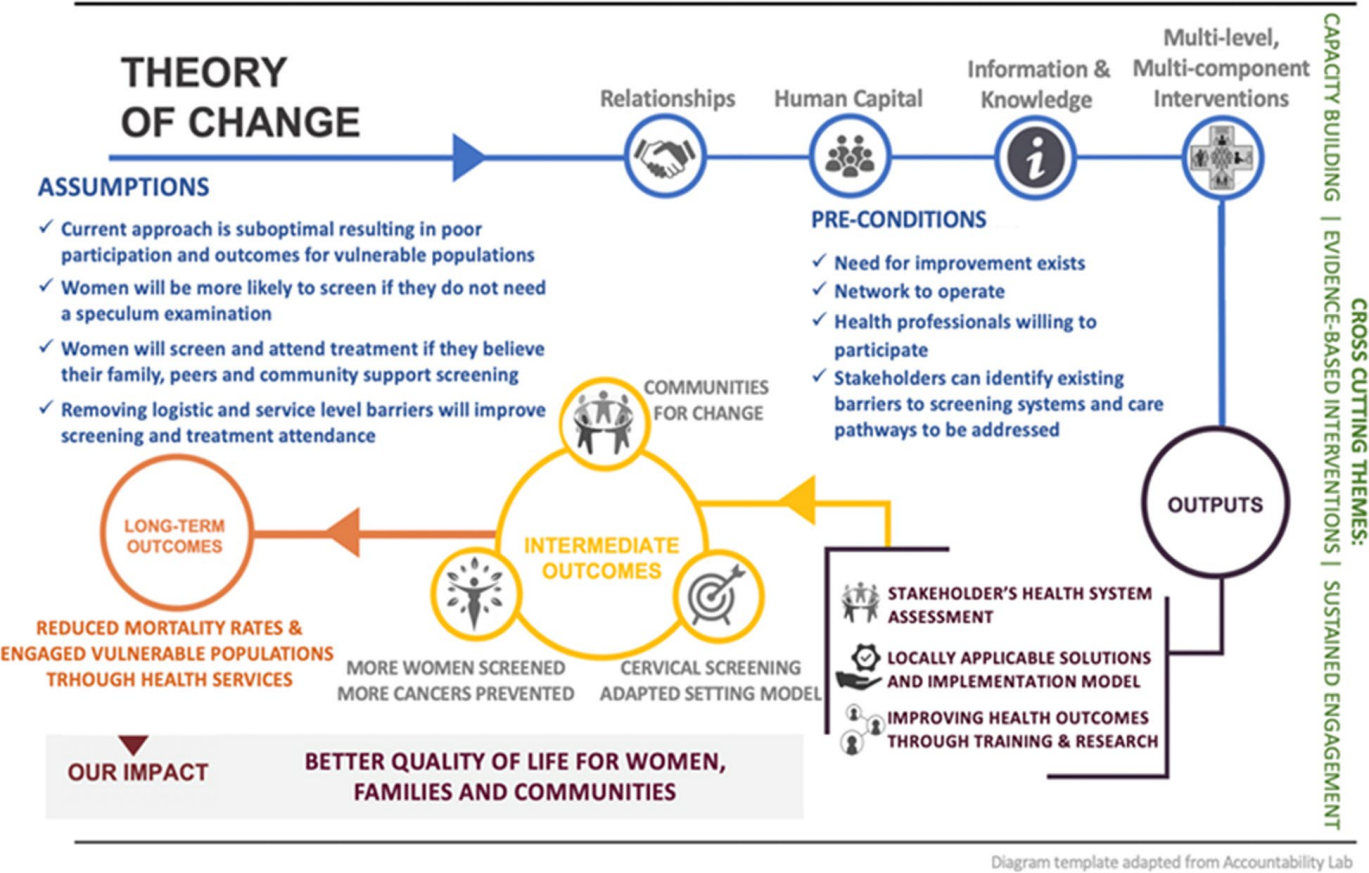
Theory of change for the SHE-CAN project

The formative phase of our study will help further develop a Theory of Change model based on a logical sequence of inputs, activities, outputs, outcomes, and possible reasons for achieving or not achieving the expected outcomes, as shown in Fig. 1.

We will utilise the Integrative Systems Praxis for Implementation Research (INSPIRE) approach [25], which explicitly incorporates current relevant implementation research theories and approaches, to evaluate scale up of HPV-based screening and treatment. The INSPIRE methodology is grounded in five approaches: systems thinking, participatory action research (PAR), soft systems methodology, the consolidated framework for implementation research (CFIR), and the RE-AIM (reach, effectiveness, adoption, implementation and maintenance) evaluation framework [26, 27]. The INSPIRE framework is centred in a model of PAR, which is designed around the principle that co-creation of interventions along with local stakeholders will result in a higher likelihood of success and more sustainable adoption. The four phases of INSPIRE will form the phases of SHE-CAN, to achieve the objectives shown in Table 1.

**Table 1:** Specific objectives of each phase and corresponding activities

Objective	Activities
Phase 1 (year 1)—Understand the system To evaluate the current status and challenges of existing cervical cancer screening services, capacity of health systems for transitioning to HPV-based screening, barriers faced by vulnerable women in accessing the cervical cancer care continuum and preferences with regards to HPV screening and treatment options	Desk review, health facility survey, qualitative studies, survival analysis of tribal women with cervical cancer vs others
Phase 2 (year 1)—Find leverage To co-design acceptable HPV-based programmatic approaches for cervical screening and treatment, for vulnerable populations, based on the findings from the formative phase,	Co-design stakeholder workshop
Phase 3 (year 2)—Act strategically To implement a cluster randomised implementation trial and evaluate implementation outcomes such as acceptability, reach and proportions well managed (screened and treated) of the HPV-based approaches, comparing to the existing VIA-based programme in India	Trial, supported by a screening registry
Phase 4 (year 3)—Learn and adapt To determine the cost effectiveness, sustainability and scale up considerations of the preferred HPV implementation model	Final stakeholder workshop

Over 3 years, we will undertake a full implementation research cycle of (1) situational analysis of ongoing VIA screening at the study sites (9 months), (2) codesigning implementation approaches for HPV-based cervical screening with or without strategies to improve the current VIA-based strategy (3 months), (3) implementation in a cluster randomised trial embedded within the existing health system (12 months), (4) evaluation of the co-designed implementation approaches, and (5) assessment of sustainability to guide ongoing implementation (12 months).

The project advisory committee and the research team include experts in screening programmes, specialist clinicians, community medicine consultants, and government, non-governmental organizations involved in cervical screening, as well as tribal health experts. In this paper, we outline the protocol for phases 1 and 2 of the study.

## Methods

### Settings

We will undertake this research at five sites, across two states of India, which reflect some of the diverse environments where vulnerable populations reside (Tamil Nadu in Southern India and Mizoram in North-eastern India). The two states selected are different in burden of cervical cancer, quality, and organisation of health systems and geo-political context.

1. Study sites in Tamil Nadu

The study will be implemented in three districts.

### Tiruvannamalai district

#### Tribal population (referred to as scheduled tribes)

Malayali (hill dwelling) tribes live in the remote Jawadhi hills. CMC Vellore offers cervical screening services to them as part of comprehensive primary care services supported by the state government. These tribal communities face the problems encountered by similar hard-to-reach tribal populations having poor healthcare access across India [28]. The study area in Tiruvannamalai district includes around 2414 women aged 30–49 years from a population of 17,686 (local census in 2022 by CMC Vellore), spread across 69 hamlets (tribal settlements). A monthly gynaecology clinic is held in CMC Vellore’s tribal primary care centre, with colposcopy, biopsy, and thermal ablation services. The study area is also served by two government Primary Health Centres (PHCs) where VIA screening is available and six Health Sub Centres (HSCs).

### Vellore district

#### Rural population

The second study site in Tamil Nadu will comprise of 65 villages in a community development block (Kaniyambadi), Vellore district (population size 87,725, local census in 2022 by CMC Vellore). The sampling frame will include 14,027 women aged 30–49 years. Population-based screening with VIA is offered by the Community Health Department, CMC Vellore, with screen positive women referred to a 140-bed secondary hospital for follow-up procedures of colposcopy and precancer management [11]. The block also has three PHCs and one Community Health Centre (CHC) with VIA screening services, as well as a Government Medical College, with facilities for follow-up of VIA positive women.

#### Urban poor population

This study site includes socio-economically disadvantaged populations in Vellore city, covered by CMC Vellore, with 11,625 population and 1577 women aged 30–49 years, across 8 wards (local census in 2022 by CMC Vellore). Community-based clinics and a secondary level centre provide opportunistic screening services through a system of volunteers, health workers, and social workers, working closely with the two local government urban health centres. There is a sub-district government hospital within this area.

### Dharmapuri district (THI, Sittilingi)

#### Tribal population

The fourth site in Tamil Nadu is the Kalrayan hills, where the THI hospital provides health care for a tribal population (Malayali and Lambadi tribes) of around 12,000 (1241 women aged 30–49 years, local census by THI) in 23 villages, as well as training of tribal girls to function as health workers and health auxiliaries. While opportunistic screening services (VIA) are available at THI hospital, cancer treatment requires referral to centres in neighbouring districts such as CMC Vellore and the government cancer centre at Kancheepuram. There is a single PHC in this area.

2. Study site in Mizoram

In Mizoram, scheduled tribes form 94.4% of the state population, the highest among all states in India [28]. The study will be implemented in Mamit district, which is the residence of a vulnerable and difficult to access rural tribal population of around 89,486 (local government census). The study area has an estimated 10,049 women aged 30–49 years, in 79 villages. The cancer screening programme is delivered through a district hospital, a CHC, nine PHCs and 39 HSCs. Cancer treatment requires referral to the Regional Cancer Centre at Aizawl, the state capital.

#### Activities planned for the study

Stakeholder engagement will contribute to all study phases, from co-designing, implementation, and evaluation through to dissemination. Stakeholders will be identified through a mapping exercise to include micro (target women, communities), meso (health professionals engaged in various levels of services, members of voluntary organisations, etc.), and macro (tribal representatives, local and state government policy makers etc.) level stakeholders. The micro level stakeholders (community representatives) at each site will advise the research group in all phases of the study, review study documents, and provide community feedback through community advisory boards in each site.

## Phase 1—situational analysis

To understand and evaluate (i) the current operation of the health care systems supporting cervical screening and treatment, including understanding the local burden of disease and community profile, (ii) availability, accessibility, affordability, and quality of screening services, and (iii) the readiness of the health care system, providers, and community members to consider implementing/participating in cervical screening using HPV testing, we will apply the RE-AIM framework [27] and the Theoretical Framework of Acceptability (TFA) [29]. This will be done using mixed methods research, among both target users and health system players with outcomes shown in Tables 2 and 3.

1. Health system evaluation

**Table 2:** Participant outcomes to be evaluated before and after the HPV-based trial

**Outcome**	**Outcome definitions**	**Current VIA programme**	**HPV trial**
**Reach**	a) Invitation rate: % invited out of eligible b) Screening participation rate: % screened out of invited c) Follow-up rate for assessment: % assessed out of screen positives d) Treatment rate: % completed treatment for pre-cancer/initiated cancer treatment, out of assessed e) Representativeness: key differences between invited vs not invited, screened and not screened, followed/treated vs those not followed up/treated f) Barriers for non-participation	Facility assessment survey checklist Qualitative study	Screening registry for trial Qualitative study
**Acceptability**	a) Acceptability of screening and associated treatment	Qualitative study	Qualitative study Feedback surveys
**Effectiveness**	a) Well managed: % of women invited who are screened and receive appropriate treatment and advice b) Number of pre cancer and cancers detected by screening and by clinical presentation c) Adverse events of screening among participants including social/economic d) Any other/unexpected health benefits associated with screening	Facility assessment survey checklist Qualitative study	Screening registry for trial Feedback surveys Qualitative study

**Table 3:** Health system-related outcomes to be evaluated before and after HPV trial

**Outcome**	**Outcome definitions**	**Current VIA programme**	**HPV trial**
**Adoption**	a) Extent to which cervical screening is available in settings in study areas b) *N*, % of settings and staff allocated to the interventions that adopted HPV intervention c) Key differences between adopters and non -adopters d) Qualitative: barriers and facilitators	Facility assessment survey checklist^a^ Qualitative study^a^	Trial evaluation of settings and providers Qualitative study^a^
**Implementation**	a) Fidelity of local implementation in comparison to national/state guidelines over time b) Nature of adaptations made and reasons c) Implementation costs	Facility assessment survey checklist^a^ Qualitative study^a^	Screening registry for trial Feedback survey Qualitative study^a^
**Maintenance**	a) Assessing sustainability of the screening programme and scale up to all areas	Trends in VIA screening coverage and frequency of offer of screening and fidelity to guidelines over time	Post-trial stakeholder assessment of sustainability and modifications, using qualitative interviews and ISAT^b^

^a^Modified IARC instruments for health facility survey and interviews [30]

^b^Intervention Scalability Assessment Tool [31]

The evaluation will include a desk review, survey of health facilities, and qualitative studies, adapting tools designed by IARC for a similar study in Europe [30].

A desk review will be done to gather information on disease burden, current screening programme guidelines/policies, screening and treatment protocols, and implementation status [30].

A quantitative survey for assessment of capacity and readiness of the health system for HPV-based screening will be done using a checklist for health facilities. This will include assessment of types of services, numbers of women screened/detected/treated for precancer or cancer, staffing, infrastructure, equipment and supplies, supply chain, governance, service charges, data systems and management (including for test results and recall methods), and community sensitisation methods.

Health facilities involved in the screening programme, such as PHCs, CHCs, and sub-district and district hospitals, as well as private organisations providing population based screening, will be identified. Simple random sampling will be done to select health facilities for capacity assessment survey if there are more than three of the same type (IARC methodology) [30, 32].

Qualitative study: Opinion of the professionals involved in organising and implementing screening and treatment in the current programme will be obtained regarding perceived barriers and opportunities to roll out HPV-based screening through semi-structured key informant interviews [33]. Findings from the capacity assessment survey and desk review will be clarified at the interviews, as needed. Current understanding of and acceptability of HPV testing including self-collection, preferences regarding offering and performing HPV-based screening and follow-up (e.g. location, organisation, staff involved, attitudes towards screen and treat programmes), and knowledge generation and identification of eligible women, as well as anticipated barriers and facilitators to implementation of HPV-based screening from the provider perspective, will also be assessed.

Those interviewed will include laboratory personnel and district programme managers, while focus group discussions (FGDs) will be conducted for community health workers such as Accredited Social Health Activists (ASHAs), ASHA supervisors, and multipurpose health workers/auxiliary nurse midwives.

2. Evaluation of target population

### Survival analysis

Given the lack of epidemiological data on stage at diagnosis and outcome of treatment of cervical cancers detected in tribal vs non-tribal women through the national cancer registries, a survival analysis will be undertaken at the Vellore subsite to confirm the suspected late stage at detection and poor survival outcomes in the former group. Women with cervical cancer seen at CMC Vellore in the previous 10 years will be identified from hospital records, noting baseline characteristics (demographic and cancer type and stage) recorded during hospital visits. Their vital status will be obtained from case files and data linkages with the death records and routine health information system maintained for the primary care service areas, supplemented where necessary by active follow-up by health workers to document current vital status. With around 31 patients from the tribal study site and 46 belonging to our non-tribal study sites (rural and urban poor women), we will have 80% power to detect a hazard ratio of 2.5, assuming 5-year survival of other groups as 70% [34].

### Qualitative study

A qualitative study will be conducted among screening-eligible women and community members to understand characteristics of screened and unscreened women, barriers to accessing screening and treatment, and unintended adverse effects or benefits associated with screening, as well as preferences related to receiving information, HPV test on self-collected samples, location for screening, receiving results, and treatment.

Semi-structured in-depth interviews will be conducted for women with lived experiences of prior screening using VIA or Pap smear (both screen positive and negative women) in each site, with the number of interviews depending on data saturation. Two FGDs with 6–8 participants each will be conducted per site (2*5 = 10), among women who have not been screened, with one of the FGDs among those who are considered most hard to reach by the community themselves (e.g. extremely poor, widowed, lower caste). FGDs for other community members who are not part of the target group (males, older and younger women) will include participants recruited through community connections/health workers, including the hardest to reach groups as a separate group. A total of 20 such FGDs are expected to be carried out across the five sites (ten each among males and females).

### Qualitative study procedures

Participants will be interviewed at a private location at a time convenient to them, by trained staff, after obtaining written informed consent. All interviews and FGDs will be audio recorded, transcribed, and translated to English (from Miso/Tamil), with all personal identifiers removed. Interviews/FGDs will be led by a trained facilitator, providing basic information about HPV-based screening, including showing the swab and instructions for self-collection. The number of interviews/FGDs will depend on overall saturation of themes raised by participants.

## Details of data analysis planned for phase 1

*Narrative synthesis of documents* reviewed will be performed, describing (1) screening programme delivery, performance, and outcomes and (2) cancer service utilisation and treatment rates for women diagnosed with cervical cancer. Programme pathways and the interaction points for patients, providers, and services will be visually mapped. CFIR domains will be used to describe the barriers and facilitators.

### Qualitative data

Transcribed data will be analysed using thematic analysis using software (NVIVO, QSR International Pty Ltd.). In addition to codes defined a priori (deductive analysis), the transcripts will be assessed to identify any emerging themes (inductive approach).

### Quantitative

Numbers and proportions of screening and treatment activities and outcomes will be reported, based on the survey of facilities. Survival rates will be estimated, disaggregated by rural, tribal, and urban-poor cohorts of women with cervical cancer diagnosed in populations covered by primary care services of CMC Vellore, with adjusted hazard ratios for predictors of survival (Cox regression) and Kaplan-Meier survival curves.

## Phase 2—co-design workshop

A stakeholder workshop will be organised following analysis of phase 1 data to:

1. Summarise and seek feedback about the results of phase 1,
2. Seek information on issues that are unclear and/or lack adequate information,
3. Share evidence-based knowledge (e.g. the evidence for self-collection, HPV-based screening and registry-based follow-up systems) and understand pros and cons of different approaches,
4. Identify leverage points for positive change, using multiple scenario analysis. These scenarios explore ‘what if’ and acknowledge that there will be pros and cons of all options,
5. Create a visual model of screening and treatment, aided by qualitatively coding the use of CFIR domains/construct terminology in the stakeholder workshops, following INSPIRE methodology [25] and
6. Obtain a shared commitment to the common goal of promoting an acceptable, feasible HPV-based screening programme.

The aim is to design HPV-based screening approaches that will best fit the local context in each type of setting, with maximal potential to impact cervical cancer incidence and mortality [25]. Key parameters that will be finalised for the trial will include where and how the offer of screening is made, where the test sample is collected, HPV assay/s type and location of testing (e.g. point of care or centralised laboratory), means of communicating the results, whether screen and treat will be used, clinical pathways for further assessment, and data items and flows for managing the implementation. Strengthening of the current VIA-based screening approach will also be considered as a possible comparator, depending on stakeholder discussions.

The steps in intervention development, followed in phases 1 and 2 of the study, will be reported according to the guidance for reporting intervention development studies in health research (GUIDED) checklist [35].

## Next steps—act strategically and evaluation

A cluster randomised trial will be done evaluating co-designed implementation approaches for HPV-based screening, in year 2 of the study, based on the arms finalised after the formative phase. The trial has been registered with the Clinical Trials Registry of India (CTRI/2022/04/042327), but exact implementation approaches/trial arms will be revised and finalised only after the initial formative phase. Hence, trial methodology will be published later. Implementation outcomes, effectiveness, and cost effectiveness, will be analysed, comparing HPV-based screening approaches to each other as well as the current VIA-based approach. Sustainability assessment will be done using the Intervention Scalability Assessment Tool (ISAT) [31] at a final stakeholder workshop.

## Discussion

WHO modelling analyses of strategies to eliminate cervical cancer as a public health problem in LMICs estimated that successful implementation at scale of girls-only vaccination, twice-lifetime HPV-based screening, with appropriate treatment of precancer and cancer, could avert 62 million deaths related to cervical cancer, in the next 100 years, with 9.9 million deaths averted in India alone [1]. What is urgently needed now is implementation research to determine exactly how countries can meet the WHO strategy’s 2030 scale-up targets for cervical screening (> 70% women aged 30–49 years are screened using a high-performance test, followed by appropriate treatment of at least 90% of cervical disease) [2]. This will require leveraging current workforce and primary health care structures and integrating the screening and treatment pathways into universal health care systems after identifying and addressing existing barriers.

Difficulty in ensuring follow-up assessment of screen-positives is a problem for any screening strategy that requires a second visit for treatment [12, 21]. An HPV-based screen and treat strategy has been found to have improved effectiveness and cost effectiveness compared to HPV testing followed by triage and treat, or VIA screen and treat strategies, in low-resource settings [36, 37], with the WHO strongly recommending it for resource limited settings [38]. However, currently, VIA, rather than HPV, is the test of choice for primary screening in the guidelines of the national programme, as well as of professional bodies, in India [39–41]. The national programme also promotes colposcopy and biopsy of VIA positive women, in spite of known challenges in scaling up of colposcopy and histopathology facilities in rural or hard-to-reach areas. Our formative phase will help inform whether an HPV-based screen and treat approach would be acceptable to women and stakeholders and which barriers are to be overcome to roll out such a programme, ensuring high participation of the tribal women and other vulnerable populations.

The protocol of the formative phase of our study illustrates the steps required to understand the system, ensuring that the concerns and needs of all stakeholders are identified prior to implementing a new programme. The health system evaluation will assess structural and dynamic fidelity of local implementation in comparison to national/state guidelines over time, and inform adaptations and changes to be implemented to support the transition to HPV-based cervical screening. Inclusion of geographically isolated tribal populations in this study, a group that constitutes 8.6% of the population of India and who face inequity in health care access and worse health indicators compared to non-tribal populations within their states, is a key strength of the study [42]. Studies reporting implementation outcomes of HPV-based cervical screening in such tribal groups are lacking in India and so is disaggregated data regarding the burden of HPV and cervical cancer mortality in such groups that are characterised by poor geographical accessibility, isolated forest or hilly location, and low education [28, 42]. One study from Udupi, Karnataka, reported 40.6% prevalence of HPV infection in tribal women aged 20–65 years [43] but did not report outcomes of follow-up and treatment, while another reported 12.9% prevalence of HPV among girls and women aged 9–25 years from central India, based on urine samples [44]. The study from Karnataka also showed lack of awareness of cervical cancer, early sexual activity, and multiple sexual partners, which could explain higher rates of HPV prevalence in even young girls [43].

Inclusion of vulnerable women from tribal groups, as well as urban slum dwellers, in our study is also in line with the Tamil Nadu State Health Policy Vision 2030 and the Tamil Nadu Health Systems Reform Program, which aim to provide focused interventions to these two groups [45].

The study brings together a team of leading health researchers and programme implementers from India and Australia, with experience in providing cancer screening and related services for vulnerable populations, along with IARC and RTI, collaborating with vulnerable communities, policymakers, and local health services. The research will also support postgraduate training opportunities using the infrastructure of the collaborating institutions, for capacity building for implementation research.

## Conclusions

Our proposed research will provide evidence and learnings to support policymakers, health providers, and others to foster successful transition of India’s cervical screening programme from a VIA-based sub-optimal quality programme to a more objective, sensitive, and scalable programme (HPV-based self-collection), in line with the 2020 WHO global strategy [38]. It will also pilot methods for ensuring that the national programme reaches vulnerable populations at highest risk in India, to ensure that elimination is implemented equitably, co-designing acceptable approaches to programme delivery that are contextualised to local needs.

## Data Availability

Not applicable.
